# Abnormal Plasma Cell Disorders in Refinery Waste Workers

**DOI:** 10.3390/jcm7080221

**Published:** 2018-08-17

**Authors:** Caterina Ledda, Carla Loreto, Vera Filetti, Serena Matera, Ermanno Vitale, Massimo Bracci, Venerando Rapisarda

**Affiliations:** 1 Occupational Medicine, Department of Clinical and Experimental Medicine, University of Catania, 95123 Catania, Italy; verofiletti@gmail.com (V.F.); serena.matera@yahoo.it (S.M.); ermannovitale@gmail.com (E.V.); vrapisarda@unict.it (V.R.); 2 Human Anatomy and Histology, Department of Biomedical and Biotechnology Sciences, University of Catania, 95123 Catania, Italy; Carla.loreto@unict.it; 3 Occupational Medicine, Department of Clinical and Molecular Sciences, Polytechnic University of Marche, I-60126 Ancona, Italy; m.bracci@univpm.it

**Keywords:** occupational medicine, multiple myeloma, industrial site, protein electrophoresis, employees, hazardous waste

## Abstract

A monoclonal gammopathy of undetermined significance (MGUS) may develop into a multiple myeloma or a correlated lymphoproliferative malignancy with a progress rate of 1% per year. The immune status, occupational-environmental risk factors, and hereditary factors may influence the risk of developing MGUS. We investigated the prevalence of MGUS in 77 refinery waste workers. They were all males, averagely aged 36, with a mean working history of 18.5 years and working in the dump for about 4.2 years. After analyzing the results of standard serum electrophoresis migrations, 16% of cases (*n* = 12) showed levels beyond the normal ranges. In all 12 samples we observed an increase of gamma component: 67%, IgG; 17%, IgM; 8%, IgA; 8%, oligoclonal. Workers were exposed to hazardous refinery waste. After the biological monitoring of urine samples for metals and t,t-muconic acid, no extra-range values were observed. The multivariate analysis shows, however, that cigarette smoking and residence near industrial sites are significantly (*p* < 0.001) associated with a high risk of MGUS development; while no association was found with occupational exposure. Additional attention might be paid in particular to these conditions in epidemiological studies and further larger, prospective, population-based researches appear warranted to evaluate the strength of any positive association.

## 1. Introduction

The plasma-cell disorders are characterized by the proliferation of a single clone of the plasma cells that produces a homogeneous monoclonal (M) protein [1,2].

In 1978, the term ‘monoclonal gammopathy of undetermined significance’ (MGUS) was established by Kyle [3]. MGUS is defined as a serum M protein <30 g/L; <10% plasma cells in the bone marrow, if done; little or no M protein in the urine; nonexistence of lytic bone lesions, anaemia, hypercalcaemia or renal insufficiency associated to the plasma cell proliferative disorder [1,3,4].

Light-chain MGUS is defined by an abnormal κ/λ free light-chain (FLC) ratio, a rise in the concentration of the concerned light chain, and an absence of expression of a monoclonal peak of immunoglobulin heavy chain in the serum on immunofixation [4,5,6].

A population-based study in the USA showed a 3.2% of prevalence for MGUS in the Caucasian general 50-years of age or older population [7].

More recently, a related study was carried out in Germany in a similar population and the prevalence detected was of 3.5% [8].

Long-term studies on MGUS cases verified an average risk of 1% per year of developing multiple myeloma (MM) or a correlated lymphoproliferative malignancy [9,10]. This risk did not reduce even after more than three decades of follow-ups [10].

More evidence of the strong correlation of MGUS with MM came from two investigations, where serially collected blood samples numerous years prior to tumor diagnosis were available [11,12]. In the last 2–3 decades, the outcomes and survival of patients with MM have significantly improved largely but nevertheless, despite advances in treatment, MM remains an incurable disease [13,14,15].

Several risk factors have been studied for MGUS: obesity, personal history of autoimmune diseases, inflammatory conditions and infections, are connected with increased risk [5].

Moreover, occupational studies have highlighted that exposure to radiation or pesticides is associated with the development of MGUS [16,17,18,19].

An increased risk of MM has been observed in workers exposed to pesticides, refinery, and refinery waste employees [20,21,22,23].

In the present study, we investigated the prevalence of MGUS in refinery waste workers and the immunoglobulin class distribution of serum monoclonal paraprotein.

## 2. Materials and Methods

### 2.1. Study Population

Between September 2017 and January 2018, 93 (100%) refinery waste workers were visited within the framework of periodic occupational surveillance and invited to participate in this study.

The inclusion criterion was having a work history in the plant of at least three years. The exclusion criteria were the use of drugs, systemic disease, such as diabetes, coronary heart disease, cerebrovascular and peripheral vascular disorders, renal disease and other blood pressure-influencing pathologies. Furthermore, we excluded workers with a history of MM, macroglobulinemia, solitary plasmacytoma, or primary amyloidosis (AL); furthermore, also patients who had a history of radiation therapy or chemotherapy were excluded.

All exposed workers were provided with a helmet, a filter mask for personal respiratory protection (fine particle mask FFP3 in accordance to EU norm EN 149), protective clothing, and gloves.

The study was performed in accordance with the guidelines of the Declaration of Helsinki and the procedures were approved by the ethical board of the University Hospital of Catania (Italy).

All workers joined the study and informed consent was obtained from all participants. Employees were interviewed by a trained occupational physician. Medical records, socio-demographic data, information about smoking habits, alcohol consumption, place of residence (i.e., living close to an industrial area) and occupational history were collected.

Long-time smokers reported their intensity of smoking (cigarettes/day) and the number of years they had smoked habitually. Pack-years exposure was determined by multiplying the duration of smoking with intensity.

### 2.2. Laboratory Analysis

Blood samples were obtained from all workers: two test tubes for serum and one with EDTA (Vacuette, Greiner Bio-One, Kremsmünster, Austria). Following the collection, the tubes for the serum were left in an upright position for at least 30 min at room temperature but no longer than 60 min. Those tubes were then centrifuged at 3500 rpm for 10 min, then the serum was isolated. The analyses were carried out on collection day.

The instruments were adjusted and internal quality control was performed using an identical lot of the manufacturer’s control and calibration material all over the study.

Haemochrome for leukocytes (WBC), erythrocytes (RBC), hemoglobin (HGB), and platelets (PLT) interpretation and biochemical parameters assay included the total protein (TP), γ-glutamyltranspeptidase (γ-GT), alanine aminotransferase (ALT), aspartate aminotransferase (AST), total cholesterol (CHOL), triglycerides (TRIG), high-density lipoprotein (HDL), low-density lipoprotein (LDL), blood urea nitrogen (BUN), uric acid (URIC), fasting glucose (GLURA) were carried out for all participants. UniCel D×H 800 Coulter Cellular Analysis System (Beckman Coulter, Brea, CA, USA) and Cobas^®^ 8000 Modular Analyzer (Roche Diagnostics, Basel, Switzerland) were utilized for haemochrome and biochemical parameters tests, respectively.

Standard serum electrophoresis migrations were performed in capillaries at 25 ± 2 °C room temperature using Minicap Sebia capillary system (Sebia, Evry, France). Any serum with an abnormal band or thought to have localized a band underwent immunofixation (IFE) (Hydrasys 2 Scan, Sebia, Evry, France) for the immunotyping of monoclonal proteins. For all analyses, the reference values proposed by the Mayo Clinic Medical Laboratories [24] were applied.

### 2.3. Waste Characterization

Waste characterization was carried out in accordance with the law [25] and any subsequent amendment [26], which provides for the classification of the waste according to Hazard Properties (HP). Three types of waste were characterized: sludge (sample A), fly ash from fuel oil (sample B) and catalysts of the Fluid Catalytic Cracking equilibrium plant (sample C).

### 2.4. Environmental Monitoring

The environmental monitoring was carried out in the refinery waste remediation plant for the quantification of inhaled airborne dust and breathable fraction [27].

The plant’s air quality was monitored bi-monthly in ten different areas with a sampling time of 240 min [28,29] and the samplings for the last three years were examined.

The sampling apparatus (Bravo B2, TCR TECORA, Milan, Italy) was placed inside the plant; all the inlet probes were positioned at 1.8 m on top of the floor (in order to simulate a human breathing zone), away from air disturbances, windows and walls. The flow rate was adjusted before and after every sampling with a bubble flow meter. The pumps were connected to filter holders containing the appropriate polycarbonate filters for inhaled airborne dust and breathable fraction (Sartorius, Gottingen, Germany).

For all samples, the total dust was determined by gravimetry using a Mettler XPE26DR micro-balance (Mettler-Toledo, Greifensee, Switzerland) and weighing was conducted in a 20 °C air-conditioned, 40% humidity room.

### 2.5. Biological Monitoring

One spot urine sample was collected from each subject at the end of an 8-h shift on day 6 of the working week. In addition, a diet was prescribed in order to not affect our study results over the whole sampling period.

Urine samples were collected in 10 mL polystyrene disposable urine collection bottles and were frozen at −20 °C until analysis.

Before proceeding with analytical determination, the urine samples were defrosted at room temperature and mixed on a rotary mixer for at least 15 min.

#### 2.5.1. t,t-Muconic Acid

t,t-muconic acid is an end product in the metabolism of benzene and is used as a biomarker for the occupational monitoring of benzene exposure [30].

t,t-muconic acid was determined by HPLC (Agilent Technologies, Santa Clara, CA, USA) with the UV detection method, using a commercial laboratory kit (Chromsystems Instruments & Chemicals GmbH, Gräfelfing, Germany).

The sample preparation is based on the efficient and selective purification with solid phase extraction. This includes the addition of a special internal standard to the sample with a simultaneous pH adjustment and subsequent transfer to the SPE column. Sequenced washing steps are then performed to eliminate interfering substances. This ensures that the method maintains high levels of precision and reliability when quantifying the analyte. Finally, the t,t-muconic acid is eluted and stabilized simultaneously. This analysis method is very sensitive and allows us to determine concentrations over 20 µg/L.

#### 2.5.2. Heavy Metal Monitoring

The urine samples were analyzed for creatinine and for arsenic (As), cadmium (Cd), mercury (Hg), lead (Pb).

All samples’ analyses were undertaken by inductively coupled plasma–mass spectrometry (ICP-MS) using an XSERIES 2 ICP-MS (Thermo Fisher Scientific, Hemel Hempstead, UK) as Goullé and colleagues described [31] and previously validated for other investigations [32].

The quality control was carried out at two levels with certificate matrices, (Seronorm^™^ Trace Elements Urine, Sero AS, Billingstad, Norway).

Urinary creatinine concentrations were calculated using a fully automated clinical chemistry analyser (Cobas^®^ 6000 Modular Analyzer, Roche Diagnostics, Basel, Switzerland) and the metal concentrations were adjusted based on creatinine levels, which were presented as micrograms per gram (µg/g) of creatinine (creu). All samples showed urinary creatinine concentrations between 0.3 and 3.0 g/L, the range suggested by the World Health Organization (WHO) as a criterion for valid spot urine samples [33].

### 2.6. Statistical Analyses

Statistical analysis was performed by the IBM SPSS Statistics 22.0 software. Normality was checked by the Kolmogorov–Smirnov test. The results were reported as the mean and standard deviation or as frequency and percentage. Multivariate logistic regression was used to explore the relations between MGUS and cigarette smoking, residence in an industrial area, duration of employment and BMI; with odd ratios (OR) and 95% confidence intervals (CI) estimated. In the logistic regression models, age was considered as a confounder. Statistical significance was set at *p* < 0.05 (two-tailed).

## 3. Results

Waste materials daily treated by workers were classified in relation to HP in three samples: sample (A) containing harmful, mutagenic and carcinogenic agents; sample (B) containing harmful, ecotoxic for reproduction, mutagenic and carcinogenic agents; sample (C) containing carcinogenic agents. Table 1 reports the characteristics detected for each sample and its main constituents.

From the environmental monitoring carried out bi-monthly for a year in 10 different plant areas, the concentration of constantly inhaled airborne dust below the threshold values, as well as a breathable fraction always below 0.05 mg/m^3^_,_ were measured (see Table 2).

Sixteen subjects out of the initially recruited 93 were ruled out from the study: 6 (38%) owing to medically treated dyslipidemia; 5 (31%) owing to medically treated hypertension; 3 (19%) owing to medically treated dysthyroidism; 1 (6%) owing to type I insulin-treated diabetes and 1 (6%) owing to type II oral hypoglycemizing treated diabetes.

The remaining 77 (83%) subjects were all males, averagely aged 36, with a mean working history of 18.5 years and working in the dump for about 4.2 years. A total of 53% (*n* = 41) were smokers and in 63 cases (82%) were living in homes close to industrial areas. Table 3 reports the sample’s main features.

After blood tests, all the levels of WBC, RBC, HBG, PLT, TP, γ-GT, ALT, AST, CHOL, TRIG, HDL, LDL, BUN, and GLURA, fell within the normal ranges (see Table 3). Likewise, exposure to biological indicators, detected through urine tests (t,t-muconic acid, As, Cd, Hg and Pb) fell within normal ranges (see Table 3).

Standard serum electrophoresis revealed that 16% of cases (*n* = 12) showed levels beyond the normal ranges. Of these, 10 (83%) lived close to an industrial area and 9 (75%) were smokers. Therefore, the IFE analysis demonstrated an IgG subtype in 8 cases (67%), IgM in 2 cases (17%), IgA in one case (8%), and oligoclonal bands in another case anomalies observed.

The band of protein migration of IFE analysis demonstrated the following anomalies: IgG in 67%, IgM in 17%, IgA in 8%, and oligoclonal in 8%. Table 4 reports the MGUS prevalence and frequency (Table 4).

Multivariate analysis for MGUS and various risk factors highlighted that cigarette smoking and residence in an industrial area were significant risk factors (*p* < 0.001); no significant association was observed for the duration of employment and BMI. Table 5 reports the results of the multivariate analysis indicating the odd ratio (OR) for each risk factor.

## 4. Discussion

The current study reports, for the first time in the literature, the data of an MGUS detected in a population of healthy workers, operating in the handling of refinery-produced waste materials. These workers are occupationally exposed to several potentially toxic, mutagenic and/or carcinogenic agents.

The percentage of MGUS-affected workers detected in our study was 16% (12 vs. 77 workers).

Usually, the MGUS produces no symptoms and is found during the laboratory test of an apparently normal patient or during assessment unrelated disorder [34,35]. MGUS is a frequent finding in the medical practice of all physicians [34,35]. MGUS occurs more often among men compared to women and the incidence is higher in blacks than in whites [36].

MGUSs have been reported in around 1% of persons older than 50 years of age and in about 3% of those older than 70 years in Sweden [37], Western France [38], and in Northern Minnesota [39]. In a recent review of the literature, Whadera and Rajkumar [35] reported that the prevalence measures of MGUS from 11 different studies ranged from 0.05% to 6.1%. In our study, patients with detected MGUS were all Caucasian, young (mean age 36), with a 16% prevalence, then higher than what was observed in other studies carried out on the general, non-occupationally exposed population [35,36,40].

In the study of the 21,463 residents of Olmsted County, the prevalence of MGUS was 3.2% [40]. The distribution of M protein isotype was immunoglobulin G (IgG) in 68.9%, IgM in 17.2%, IgA in 10.8%, and biclonal in 3.0%. The serum light-chain type was kappa in 62% and lambda in 38%. The M-protein concentration was <10 g/L in 63.5%, 10–14.9 g/L in 16.6%, 15–19.9 g/L in 15.4%, and ≥20.0 g/L in 4.5%.

In our study, the M protein isotype was comparable to what reported by Kyle et al. (2004) [40], in particular: IgG in 67%, IgM in 17%, IgA in 8%, and oligoclonal in 8%. In MGUS patients it is important to know whether the M protein will remain stable and benign or progress to MM or a related disorder [1,34].

Some patients affected by MGUS may develop symptomatic MM, Waldenstrom macroglobulinemia (WM), AL, or a related monoclonal plasma cell proliferative disorder for the period of follow-up [1,34].

Whadera and Rajkumar [35], in a literature review, reported that the rate at which MGUS progresses to MM or a related disorder is 1% per year [41,42]. The main risk factors for progression of clinical MGUS are size and type of serum M protein and the presence of an abnormal serum free light chain ratio [43]. Patients with an IgM or IgA paraprotein have a significantly increased risk of progression compared to individuals with an IgG protein [9]. Bladé et al. [44] also reported that patients with an IgA MGUS had a superior probability for a progression to MM.

In our sample, 25% of subjects showed an IgM (17%) and IgA (8%) component, but none of them showed symptoms or signs of pathologies or any other blood alterations. In a series of 1104 patients with MGUS, >5% bone marrow plasma cells, the presence of Bence Jones proteinuria, a polyclonal immunoglobulin decrease, and an elevated erythrocyte sedimentation rate were independent factors influencing the evolution of MGUS [45].

In the subjects of the present study, no Bence Jones proteinuria, polyclonal immunoglobulin reduction and/or elevated erythrocyte sedimentation were observed. Amongst the mutagenic/carcinogenic agents that these workers were exposed to were heavy metals (As, Cd, Hg, and Pb) and benzene. After the biological monitoring of urine samples for As, Cd, Hg, Pb, and t,t-muconic acid, no extra-range values were observed. Benzene exposure is a recognised risk factor for MM [46]. Evidence for an association between exposure to benzene and MM has been provided by two population-based case-control studies that reported increased MM risk ratio with rising exposure to benzene [47,48]. In our workers, the values of biological exposure indicators were comparable to those found in the general non-exposed population, despite a positive tabagic habit.

Little is known about the aetiology of MGUS. The prevalence increases drastically with advancing age and is higher among males and blacks [13,32]. Immune status and occupational, environmental, and genetic factors may influence the risk of developing MGUS [35,49].

In our sample, 82% (*n* = 63) of subjects resided close to industrial plants. Patients with MGUS are also at risk of correlated disorders, such as light-chain amyloidosis and macroglobulinemia. Conditions such as osteoporosis, hip fractures, and peripheral neuropathy are also correlated with MGUS [50].

In our study sample, given also the young age, no other pathologies were associated.

An increased incidence of MGUS is recognized in immunosuppressed and/or immunocompromised patients [17,51,52,53]. A connection between MGUS and human immunodeficiency virus (HIV)-seropositive (immunocompromised) patients has been reported, but this relationship has not been well defined. Patients infected with HIV have a risk of MGUS that ranges from 9% to 45%, considerably higher than that of HIV-seronegative patients of the same age [17,51,52,53]. Several studies also recommend that monoclonal gammopathy occurs at a younger age in those infected with HIV than in the general population [51,54,55].

The incidence of MGUS is also higher in patients receiving forms of immunosuppressive treatment [35]. In our study, none of the subjects were immunosuppressed or showed visibly infectious pathologies.

Confirmation pertaining to the influence of environmental factors on risk of MGUS has been contradictory overall. Though, a higher risk of MGUS has been confirmed in farmers, industry employees, and those with a history of occupational or environmental exposure to toxins [38]. Occupational exposure to asbestos, aromatic hydrocarbons, fertilizers, mineral oils and petroleum, paints and connected products, pesticides and radiation has been shown to be significantly associated with an increased risk of MGUS [56,57]. In addition, a link also exists between heavy smoking practice and various hematologic conditions, including MGUS [11,57].

In our sample, 53% (*n* = 41) were smokers with an average consumption of 301 cigarette packs/year; besides, 63 subjects (82%) were living near industrial plants. A multivariate analysis seems to show that cigarette smoking and residence near industrial sites were significantly associated (*p* < 0.001) with a high risk of MGUS development, while no association was found with occupational exposure.

Numerous small-scale reports advise that, in some cases, familial predisposition to MGUS may be increased [58]. Familial aggregation of MM has been documented for years [6]. Lately, a large population-based study in Sweden demonstrated a 2-fold increased risk of multiple myeloma among first-degree relatives of patients with MM [12]. In the similar investigation, there was evidence of an increased risk of MGUS between relatives of patients with MM. In addition, a prospective study found that the relative risk of MGUS in first-degree relatives of an MGUS proband was 3-fold higher than in the general population [59].

From the medical history of each worker, no family records for MM were reported. However, in 9 cases (%) a positive familial medical history was observed as to neoplastic pathologies, namely, 4 cases of breast cancer, 2 cases of lung cancer, 1 case of colon cancer, 1 case of thyroid cancer, and 1 case of pancreas cancer.

Although the causes of MGUS and MM remain largely unclear, the previous cohort [21,41,42,43,60,61,62,63] and case-control studies [64,65,66,67,68,69,70,71,72,73,74,75,76,77] have reported a superior risk of MM between farmers and other agricultural employees. The prevalence of MGUS among male pesticide applicators from the Agricultural Health Study (AHS) was found twice as much as in a population-based sample of men from Olmsted County (MN, USA) [11]. More particularly, pesticides (i.e., insecticides, herbicides, fungicides) have been hypothesized as the basis for these relations [48,78,79,80]. In the first prospective cohort study estimating MGUS risk in relation to pesticide exposure in a sample of 678 male pesticide applicators, a 2-fold significantly increased prevalence of MGUS was observed between pesticide applicators, adding support to the hypothesis that pesticides are linked to myelomagenesis [11].

Only in 3 cases (4%) was it possible to detect the use of pesticides in the spare time due to the cultivation of small pieces of private lots.

To this purpose, a recent study conducted on 958 U.S. Air Force (USAF) military workers who conducted aerial herbicide spray missions (Agent Orange: 2,4-dichlorophenoxyacetic acid, 2,4,5-trichlorophenoxyacetic acid, 2,3,7,8-tetrachlorodibenzo-p-dioxin (TCDD)) in Vietnam, from 1962–1971, supports the hypotesis that certain pesticides play a role in the development of MGUS [11]. In fact, the odds of MGUS increased with increasing body burden of TCDD [16].

As already described by Kyle and Rajkumar (2007) [1], the management of MGUS patients provides that the serum protein electrophoresis is repeated after 3–6 months to exclude MM. If the results are constant and the patient shows no clinical features of MM, WM, or AL and a serum M protein value <15 g/L IgG type and normal FLC ratio, serum protein electrophoresis may be repeated every 2–3 years. In patients with a serum M protein value >15 g/L, IgA, or IgM protein type, or an abnormal FLC ratio, a bone marrow aspirate and biopsy should be performed.

Then, the sanitary surveillance of these subjects, which is law regulated in Italy, will be carried out in order to have a strict control of these workers and to evaluate any onset of clinical signs and symptoms which may require specific diagnostic analysis [81,82,83].

On the basis of our supplementary literature review of the evidence, the immune status, environmental risk factors and hereditary factors may influence the risk of developing MGUS. MGUS occurs more frequently in those who are immune-compromised, such as patients who are infected with HIV or who have undergone a transplant [11,51,52]. Exposure to toxins, including asbestos, fertilizers, and pesticides, appears to increase the likelihood of developing haematological disorders including MGUS [84,85,86]. In conclusion, first-degree relatives of those affected by MGUS are at a 3-fold higher risk of having the disease, raising the possibility of shared genetic or environmental factors [35,59].

In the present investigations, we observed for the first instance asymptomatic workers with MGUS.

From the medical histories and clinical-laboratory analyses, there is no proof of haematological pathologies in the family members of these subjects, nor even immune status alterations. However, smoking habits have been observed in 9 (75%) workers, and in all subjects with MGUS, and positive feedback in 82% of the sample (*n* = 63), as well as in all MGUS patients residing in proximity of industrial plant areas. From the results of multivariate analysis, both cigarette smoking and the place of residence demonstrated a significant (*p* < 0.001) association with MGUS, while no association was found with occupational exposure.

The limits of this study are the small number of the sample under examination and the small number of years of exposure.

## 5. Conclusions

MGUS is one of the most frequent premalignant disorders in the general population. It is clear that epidemiological studies will aid in the detection of groups at a high risk for MGUS and thus at a high risk for the subsequent progress to MM. This study provides additional information on the risk factors for MGUS, indicating an association with cigarette smoking and residence close to industrial sites, though the occupational exposure, which is particularly contained and monitored in the various work environments, does not seem to be associated with the risk for MGUS development. Additional attention should be paid in particular to these conditions in epidemiological studies and further larger, prospective, population-based research appears warranted to assess the strength of any helpful association.

## Figures and Tables

**Table 1:** The waste characterization.

Sample	Hazard Properties (HP)
A	HP 5 “Harmful”: waste which can cause specific target organ toxicity either from a single or repeated exposure, or which cause acute toxic effects following aspiration. HP 7 “Carcinogenic”: waste which induces cancer or increases its incidence. HP 11 “Mutagenic”: waste which may cause a mutation, that is, a permanent change in the amount or structure of the genetic material in a cell.
B	HP 5 “Harmful”: waste which can cause specific target organ toxicity either from a single or repeated exposure, or which cause acute toxic effects following aspiration. HP 7 “Carcinogenic”: waste which induces cancer or increases its incidence. HP 10 “Toxic for reproduction”: waste which has adverse effects on sexual function and fertility in adult males and females, as well as developmental toxicity in the offspring. HP 11 “Mutagenic”: waste which may cause a mutation, that is, a permanent change in the amount or structure of the genetic material in a cell. HP 14 “Ecotoxic”: waste which presents or may present immediate or delayed risks for one or more sectors of the environment.
C	HP 7 “Carcinogenic”: waste which induces cancer or increases its incidence.

**Table 2:** The environmental monitoring.

Area	Inhaled Airborne Dust (mg/m^3^)		Breathable Fraction (mg/m^3^)	
1	0.08 ± 0.02	TLV-TWA 10 mg/m^3^	<0.05	TLV-TWA 3 mg/m^3^
2	0.10 ± 0.02		<0.05	
3	0.06 ± 0.01		<0.05	
4	0.06 ± 0.01		<0.05	
5	0.08 ± 0.02		<0.05	
6	0.06 ± 0.01		<0.05	
7	0.08 ± 0.02		<0.05	
8	0.06 ± 0.01		<0.05	
9	0.08 ± 0.02		<0.05	
10	0.06 ± 0.01		<0.05	

**Table 3:** The individual characteristics and laboratory results in observed workers.

Workers (No. 77)	Mean ± SD	Reference Values
General characteristics		
Age (years)	36 ± 11	
Duration of employment (years)	18.5 ± 6.4	
Duration of employment in refinery waste (years)	9.1 ± 3.9	
Smoking habits	41 (53%)	
Pack/years	301 ± 36	
Living close industrial area	63 (82%)	
BMI (Body Mass Index) (kg/m^2^)	22.1 ± 2.5	18.5–24.99
Hematology		
WBC (Haemochrome for leukocytes)	7.3 ± 1.7	4–10 × 10^3^/µL
RBC (erythrocytes)	5.1 ± 1.6	4–6 × 10^6^/µL
HGB (hemoglobin)	15.4 ± 1.2	13.0–17.5 g/dL
PLT (platelets)	251 ± 74	150–450 × 10^3^/µL
Biochemical parameter		
TP (total protein)	6.9 ± 0.5	6.3–7.9 g/dL
γ-GT (γ-glutamyltranspeptidase)	21 ± 12	9–40 U/L
ALT (alanine aminotransferase)	25 ± 15	7–55 U/L
AST (aspartate aminotransferase)	26 ± 18	8–48 U/L
CHOL (total cholesterol)	187 ± 23	<200 mg/dL
TRIG (triglycerides)	95 ± 26	<150 mg/dL
HDL (high-density lipoprotein)	63 ± 12	≥40 mg/dL
LDL (low-density lipoprotein)	110 ± 18	≤129 mg/dL
BUN (blood urea nitrogen)	15 ± 5	8–24 mg/dL
URIC (uric acid)	5.9 ± 2.1	3.7–8.0 mg/dL
GLURA (fasting glucose)	95 ± 16	70–126 mg/dL
Biological Monitoring		
t,t-muconic acid	278.8 ± 78.3	500 µg/L
As	24.3 ± 8.9	<50.0 µg/g creu
Cd	1.2 ± 0.8	<3.0 µg/g creu
Hg	19.7 ± 3.5	<35.0 µg/g creu
Pb	3.1 ± 1.7	<5.0 µg/g creu

**Table 4:** The MGUS (monoclonal gammopathy of undetermined significance) Prevalence and Frequency of Ig sub-types.

MGUS Prevalence No. (%)	Frequency of Ig Sub-Types No. (%)				
	IgG	IgA	IgM	IgD	Oligoclonal
12 (16%)	8 (67%)	1 (8%)	2 (17%)	0	1 (8%)

**Table 5:** The multivariate analysis of MGUS risk factors.

	MGUS	
	OR (95%CI)	*p*-Value
Duration of employment	1.04 (0.91–1.17)	n.s.
Duration of employment in refinery waste	0.99 (0.95–1.04)	n.s.
Smoking habits	1.08 (1.05–1.13)	<0.001
Living close industrial area	1.10 (1.07–1.15)	<0.001
BMI	1.03 (0.97–1.09)	n.s.

n.s.: not significant.
